# Length weight relationships of coleoid cephalopods from the eastern Mediterranean

**DOI:** 10.1038/s41598-022-16611-7

**Published:** 2022-07-18

**Authors:** Bahadır Önsoy, Alp Salman

**Affiliations:** 1 Faculty of Fisheries, Muğla Sıtkı Koçman University, 48000 Muğla, Turkey; 2 Faculty of Fisheries, Ege University, 35100 Bornova, Izmir Turkey

**Keywords:** Marine biology, Invasive species, Population dynamics, Invasive species, Population dynamics

## Abstract

Length-weight relationship (LWR) studies have been widely conducted for fish. They are important because they provide information about the growth of the fish, its general wellbeing, and fitness in a marine habitat. In comparison, relatively few LWR studies have been conducted on cephalopods. A total of 13,474 specimens belonging to 28 cephalopod species was investigated to define their length–weight relationship status and Fulton’s condition factors, and compared with previous studies to evaluate life history traits and test comparability of LWR values. Isometry was found in 8 species including 2 teuthids, 2 sepiids and 4 octopods, and positive allometry was found in 2 squid species. Other species showed negative allometry. Four orders of the class Cephalopoda distributed in the Mediterranean Sea were also compared in respect of their coefficient *b* values, and a clear distinction was found between the orders reflecting their characteristic body types and thus lifestyles. Coefficient *b* values of mature animals were found lower than that of maturing ones that reflects growth of semelparous cephalopods stops or at least slows down when they reach maturity. Some extreme condition factor values were calculated for especially octopods that one of them reached to 140.91 in a deep-sea octopus *Pteroctopus tetracirrhus.* It suggests that there are many factors that might affect the calculations. Some of them were: different body structure and growth type in cephalopods than that of fish, different length measurement method applied in cephalopods, different body parts that might have different growth rates, and preservation methods that could affect the body shape and weight in soft bodied animals.

## Introduction

Organisms increase in size (i.e. length and weight) as they develop. Aside from size, age and sexual maturity, the key factors that influence how much an individual grows includes the amount of food available, the number of competitors using this food source, as well as environmental factors such as temperature, oxygen and other water quality factors. Therefore, length–weight relationships (LWR) and Fulton’s condition factor (K) is widely used in fisheries and fish biology studies^1–3^ to find out how environmental factors affect the animals’ sizes for better and sustainable fishery management by applying stock assessment models, which directly uses LWR data. The weight of organisms is exponentially related to their length, and the slope (coefficient *b*) of the relation between length and weight indicates the shape and growth of the organism^4^ (i.e. “isometric” when *b* = 3, “negative allometric” when *b* < 3, and “positive allometric” when *b* > 3).

Although LWR is widely used and known for fish, it is poorly understood for non-fish species^5^. In fact, cephalopods particularly should be investigated carefully for LWR studies as they have unique life history traits such as semelparity, sexual dimorphism (extreme in some species e.g. pelagic octopods), rapid growth in relatively short life time (the most species have around a year life time) etc.^6,7^. Cephalopod growth has two phases that could be split into rapid, “exponential” growth phase at the initial part of the animal’s life, and so called “logarithmic” growth phase at second part^8^. Unlike in many fish species, cephalopods do not have larval phase. Also, different body parts of a cephalopod might have different growth rates^9^. Furthermore, because all coleoid cephalopods die after the reproduction ends (i.e. semelparity), many of them stop feeding during the mating/spawning event^6^, thus this may affect the animals’ condition. These factors could make LWR studies on cephalopods difficult in comparing results of different studies unlike that of fish, so sampling of a LWR study should better cover whole life cycle of a cephalopod species studied.

A total of 54 cephalopod species have been reported from the eastern Mediterranean Sea^10^. These species belong to 4 orders (Sepiida, Sepiolida, Teuthida, and Octopoda) and include Lessepsian migrants *Sepioteuthis lessoniana*, *Sepia pharaonis* and *Octopus aegina.* They also include species of commercial value such as *Loligo vulgaris, Illex coindetii, Octopus vulgaris, Sepia officinalis,* etc. Since LWR data and conversion factors might reflect the condition of the populations, monitoring those data and comparing them in the populations from different habitats could be very important for better fishery management and to estimate the possible effects of the invasion (e.g. Lessepsian migration) where applicable.

This study aims contributing to increase knowledge on LWR of cephalopods as well as comparing them with other studies to understand whether there are spatial or temporal differences by analysing large numbers of cephalopod samples collected from eastern Mediterranean Sea in last three decades to reduce errors caused by special biological characters of cephalopods mentioned above.

## Materials and methods

A total of 13,474 specimens belonging 9 families and 28 species was collected in different dates and studies between 1991 and 2017. From 1991 to 1993, 1996 to 1998, and 2007 to 2008 the samples were collected by research cruises conducted by RV/K. Piri Reis by trawl operations [the samples were kept in authors’ personal collection for further uses under the permissions of the managers of the research cruises. Later on, some of the samples moved to Ege University Faculty of Fisheries Museum (ESFM)^11^]. Between 2016 and 2017, 95 out of 238 specimens of *Octopus vulgaris,* and between 1999 and 2001, 638 out of 755 specimens of *Sepia officinalis,* and between 2012 and 2014, 248 out of 483 specimens of *Loligo vulgaris* were gathered from fishermen. All samples were collected randomly by authors themselves, and the study was conducted on dead animals (Because of the nature of trawl operations and preparation process of trawl contents by seamen, all samples reached the authors were already dead). Dorsal mantle lengths (DML) and total body weights (TW) were taken to the nearest 0.1 cm and to the nearest 0.01 g, respectively either on board from fresh material (only samples of *O. vulgaris* were investigated upon fresh animals) or at the laboratory after preserving 10% formalin solution. Samples did not include juvenile specimens.

Length–weight relationships were calculated using the formula *W* = *aL*^*b*^ where *W* is the total weight (in grams), *L* is the DML (in centimetres), and *a* and *b* are the parameters of the equation. The standard error of the slope (SE*b*) and the correlation of coefficient (R^2^) were also calculated. One way ANOVA was applied to see whether there are differences in *b* calculated amongst orders (p > 0.05).

Fulton’s condition factor formula is *K* = 100 × *W/L*^3^ where *K* is Fulton’s condition factor, *W* is the weight (in grams), and *L* is dorsal mantle length (DML, in centimetres).

Calculated coefficient *b* values were evaluated according to general assumption i.e. “isometric” when *b* = 3, “negative allometric” when *b* < 3, and “positive allometric” when *b* > 3.

In order to evaluate the changes in body condition and shape at maturity which assumed as waypoint, because cephalopods have two growth phases in their lifetime as mentioned before, maturing and mature animals’ coefficient *b* values of ten species (that of reproduction biology data on maturation of the population available from previous studies: *Sepia officinalis*, *S. elegans*, *S. orbignyana, Sepietta oweniana*, *Rossia macrosoma*, *Illex coindetii*, *Loligo vulgaris*, *Alloteuthis media*, *Octopus vulgaris*, and *Eledone moschata*) were compared by splitting each species into two groups by the point of DML at which 50% of the population reaches maturity (Table 1). There was no mature females of *Octopus vulgaris* and *Eledone moschata* within our data thus, separation the females into maturing and mature is not applicable. Because of the fact that cephalopods have two different growth phases i.e. their growth turns logarithmic increase from exponential growth, so maturity can be taken as a waypoint.

**Table 1:** Dorsal mantle lengths (in mm) of the species at which 50% of the population reaches maturity (*N/A* not applicable).

Species	Female	Male	References
*Sepia officinalis*	100	90	Önsoy and Salman^12^
*Sepia elegans*	41	42	Salman^13^
*Sepia orbignyana*	70	50	Dursun et al.^14^
*Sepietta oweniana*	28	24	Salman^15^
*Rossia macrosoma*	40	30	Salman and Önsoy^16^
*Illex coindetii*	164.8	139.3	Salman^17^
*Loligo vulgaris*	165	130	Mangold-Wirz^18^
*Alloteuthis media*	36	31	Salman^19^
*Octopus vulgaris*	N/A	150	Unpublished data
*Eledone moschata*	N/A	80	Önsoy and Salman^20^

Authors hereby declared that all methods carried out in accordance with EU Directive 2010/63/EU for the care and welfare of animals used for experimental and/or other scientific purposes.

### Ethical approval for research involving human participants and/or animal

This article does not contain any studies with human participants performed be any of the authors. This study is involved with invertebrate animals. However, cephalopods have been included in EU guidelines for the care and welfare of animals used for experimental and/or other scientific purposes in Directive 2010/63/EU in 2010. Although this study includes cephalopods collected in 30 years (i.e. before inclusion to the EU Directive), all applicable international, national, and/or institutional guidelines for the care and use of animals were followed.

### Informed consent

The authors declare that the study does not have any individual participant that requires inform consent.

## Results

To evaluate the length–weight relationships of the eastern Mediterranean cephalopods, the species, sample size (in numbers), mantle length range (cm, DML), total weight range (g, TW), length–weight parameters *a* and *b*, the standard error of the slope (SE*b*) and the correlation coefficient (R^2^) were measured/calculated and are given for each sex of a species in Table 2 in Supplementary Material. Calculations of just one sex were given for some species, because of the sampling gears’ selectivity for extremely small sizes of individuals or the natural rarity of the species (i.e. pelagic octopods, some sepiolid species). Therefore, abbreviations *F* (for females) and *M* (for males) and *B* (for both sexes) were mentioned after the species name in parenthesis where needed to refer that the result represented the relative sex.

**Figure 1:**
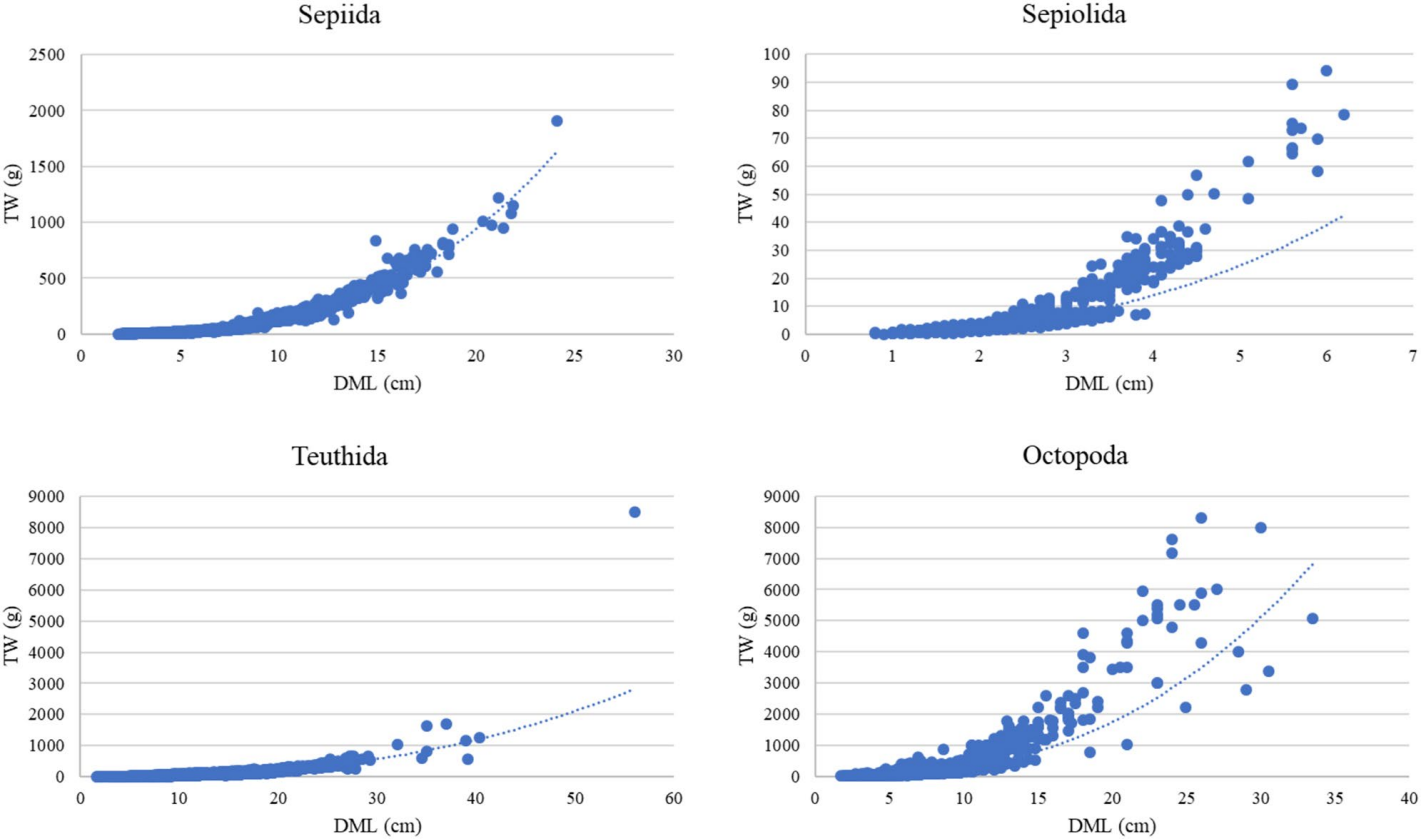
Length–weight distribution of cephalopods by orders (*TW* total weight in grams, *DML* dorsal mantle length in centimetres).

**Figure 2:**
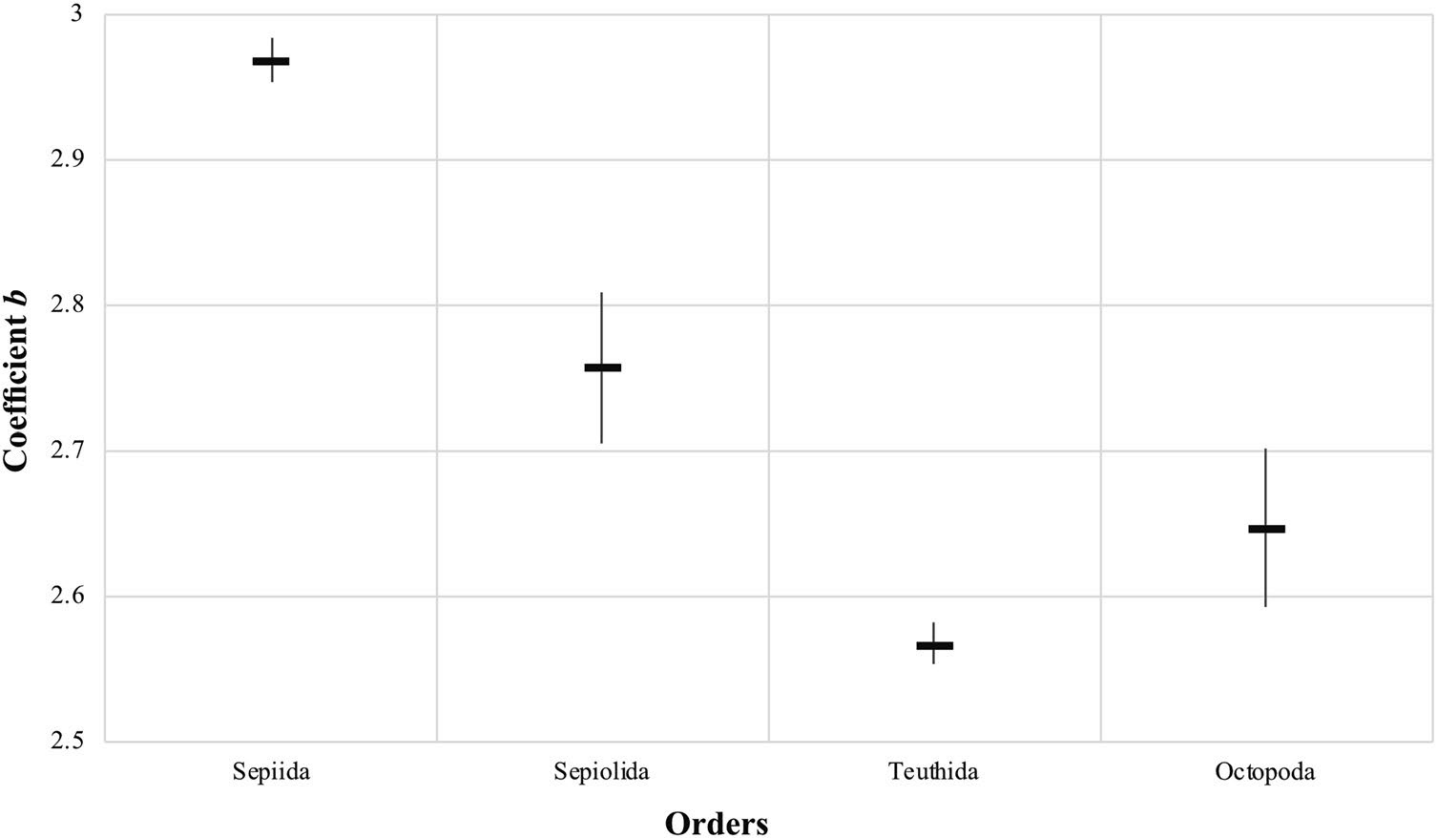
Coefficient *b* values comparison between orders with standard deviation ranges.

The mantle lengths varied between 1.9 and 24.1 cm (mean 6.12 ± 2.91 cm; n: 3930) in the order Sepiida, 0.8 and 6.2 cm (mean 2.25 ± 0.74 cm; n: 1975) in the order Sepiolida, 1.6 and 56.0 cm (mean 8.01 ± 5.19 cm; n: 5986) in the order Teuthida, and 1.7 and 33.5 cm (mean 8.15 ± 3.57 cm; n: 1583) in the order Octopoda (Fig. 1). Also, coefficient *b* values were calculated for orders as follow respectively, 2.968 (± 0.015), 2.757 (± 0.052), 2.567 (± 0.014), and 2.647 (± 0.054; Fig. 2).

Isometry is indicated by the value of three of the length–weight parameter *b,* values other than three indicate allometric growth as in fish^21^ but this is also applied in non-fish species^5,22^. There were 8 species (*Todarodes sagittatus* (*F*), *Abralia veranyi* (*M*), *Sepiola robusta* (*F*), *Sepiola steenstrupiana* (*M*), *Octopus vulgaris* (*B*), *Ocythoe tuberculata* (*F*), *Tremoctopus violaceus* (*F*), *Octopus aegina* (*M*)) showed isometry out of 28 species. Only *Todarodes sagittatus* (*M*; *b* = 3.367, SE*b* = 0.170 and *B; b* = 3.207, SE*b* = 0.113) and *Ommastrephes bartramii* (*F*; *b* = 3.406, SE*b* = 0.219 and *B; b* = 3.405, SE*b* = 0.205) showed positive allometry. Other species had negative allometry where parameter *b* ranged between 1.159 and 2.948. In squids (order: Teuthida), the parameter *b* varied from 1.97 to 3.11 (mean: 2.52 ± 0.37), in Sepiida from 2.36 to 2.82 (mean 2.56 ± 0.23), in Sepiolida from 1.36 to 3.18 (mean 2.23 ± 0.64), and in Octopoda from 1.68 to 2.99 (mean 2.36 ± 0.37; see Table 2 in Supplementary Material). There was no significant difference in *b* found amongst the orders (One way ANOVA, p = 0.6645). *a* and *b* values reported by previous studies from the Mediterranean Sea are also given in Table 3 (see Supplementary Material) for comparison.

Fulton’s condition factors (K) varied from 3.02 to 31.31 (mean 12.51 ± 2.61) in Sepiida, from 9.16 to 125.55 (mean: 31.03 ± 12.48) in Sepiolida, from 0.95 to 36.64 (mean: 4.48 ± 2.13) in Teuthida, and from 7.39 to 250.50 (mean 35.47 ± 24.91) in Octopoda (see Table 2 in Supplementary Material).

**Figure 3:**
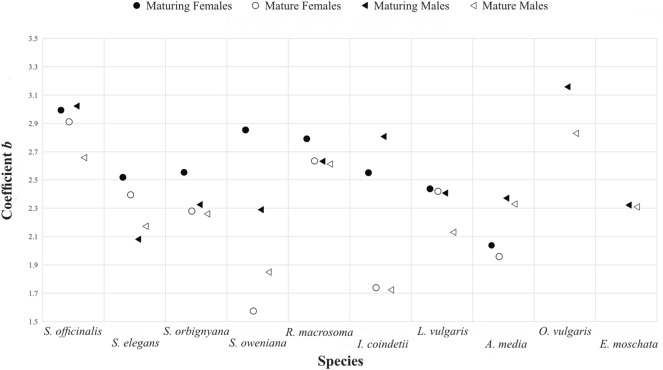
Comparison of coefficient *b* values of maturing and mature animals.

Coefficient *b* values of mature animals found lower than that of maturing ones in all species investigated (except in *Sepia elegans* males; M_mature_ *b* = 2.173 and M_maturing_ *b* = 2.081; Fig. 3).

## Discussion

Length–weight relationships of non-fish species naturally bear some difficulties on evaluating the outcome values because of the body type of the animals. Furthermore, cephalopods have different body parts that may have different growth rates i.e. arms, head and mantle that influence the weight value so makes difficult applying the terms isometry and allometry in relation to the slope coefficient unlike in fish^9^. However, using the terms isometry and allometry makes it clear to understand the animals’ body shape and differences or similarities between the cephalopod species, not exclusively for indicating growth type. There are two different growth phases described i.e. exponential, which is initial rapid growth and logarithmic, which indicates slower growth for cephalopods individually^8^. Therefore, a coleoid cephalopod species (all animals from subclass Coleoida which was subjected to this study are semelparous) has an early rapid growth phase and then a decreasing growth rate gradually after reaching maturity.

### Sepiida

Four species belonging to Sepiida are distributed in eastern Mediterranean Sea (*Sepia officinalis, S. elegans* and *S. orbignyana, S. pharaonis*). Three species (except *S. pharaonis*) investigated in this study showed negative allometry with *b* values ranged between 2.159 (in *S. elegans* (*M*)) and 2.875 (in *S. officinalis* (*F*)). As reported by previous studies from whole Mediterranean Sea^23–28^, they showed negative allometry as found in this study except one from eastern Mediterranean Sea (*b* = 3.16 for *S. officinalis* by Akyol and Metin^26^). There are great variances between the *b* values given for different species by different studies as seen in Table 3 in Supplementary Material, possibly caused by temperature and food availability or sampling methodology.

### Sepiolida

There were 6 species were investigated in this study and just for one species’ (*Sepietta oweniana*) LWR reports found to compare^27,29^. All sepiolids studied were found negative allometric except *Sepiola robusta* ((*F*) *b* = 3.623) and *S. steenstrupiana* ((*M*) *b* = 3.175) that showed isometry. *b* values reported by previous works on *Sepietta oweniana* varied between 1.61 and 1.97 for females^27,29^. Giordano et al.^29^ also reported *b* value for males of *S. oweniana* as 1.29. In this study, *b* values were found 2.53 for females, 1.92 for males, and 2.25 for both sexes in *S. oweniana*. There are great differences in given *b* values between the studies might be seen for *S. oweniana* as well as appeared for sepiids by the similar reasons explained above (see Table 3 in Supplementary Material).

### Teuthida

All squids investigated except *Todarodes sagittatus, Ommastrephes bartramii* and *Abralia veranyi* showed negative allometry. *b* values given by different studies of *Alloteuthis media, Loligo forbesi, Illex coindetii*,* Todaropsis eblanae* and *Todarodes sagittatus* were found similar to each other and to this study whereas that of *Loligo vulgaris* had variances with Duysak et al.^28^, probably caused by difference in sampling period and/or selectivity of the gears used (see Table 3 in Supplementary Material).

### Octopoda

Eleven species out of 28 in this study belonged to the order Octopoda, and 6 of them (*Octopus vulgaris, Eledone cirrhosa, E. moschata, Pteroctopus tetracirrhus, Octopus salutii* and *Bathypolypus sponsalis*) were found to be compared with previous studies. Benthic octopods *O. vulgaris* and *O. aegina,* and pelagic octopuses (*Tremoctopus violaceus, Ocythoe tuberculata* and *Argonauta argo*) showed isometry; others showed negative allometry except another pelagic octopus *Argonauta argo* that we had insufficient numbers of sample to calculate LWR. Because of the enormous size differences between the sexes of pelagic octopods, there were no male samples that are extremely small in sizes, could be caught and investigated. Also, similar problem occurred for *B. sponsalis* males and *O. aegina* females because of their rarity. There are variances between the *b* values reported by previous studies (see Table 3 in Supplementary Material).

There were some extreme K factors found especially in the order Octopoda (e.g. *Pteroctopus tetracirrhus* K: 140.91, *Scaeurgus unicirrhus* K: 82.79), which was another problem we encountered. It might be called “preservation effect” that formalin’s shrink effect of exposed tissue^30^. This might not be a big issue for whom study on vertebrates and/or animals have hard skeleton but it could make huge errors on calculating LWR when soft bodied animals are the subject. Also, it was caused by the animals’ different sized body parts (i.e. arms) which were not taken into consideration while measuring the length (in general dorsal mantle length is measured of cephalopods). We argue that the reason for extreme values are related with both issues.

There is a clear distinction was found between the *b* values of orders that represents different characteristics of body shapes for each. According to the assumption of coefficient *b* departs from 3.0 to the extent that a cephalopod is not spherical^9^, we assumed that the lower b value out of 3.0 the slimmer the animal. Squids have more torpedo-like body shape than others thus the *b* value was found the smallest (2.567), whereas that of cuttlefish was the largest (2.968) as they have thick CaCO_3_ cuttlebone that might increase the body weight. Sepiolids and octopods on the other hand, have long arms/short mantle lengths that affect the length–weight ratio, thus it put their position in between Sepiida and Teuthida in respect of the coefficient *b.* Also, it might be evolutionary response to different lifestyles, i.e. fast swimmer, pelagic ones have more hydrodynamic body type than the demersal and/or benthic species.

Coefficient *b* values of mature animals were found lower than that of maturing animals (Fig. 3). This might be caused by feeding stops in reproduction season of many cephalopod species^6^. Also, this phenomenon could be seen in previous studies on growth of cephalopods that reported as cephalopods have two different growth phases; an initial rapid growth phase and latter logarithmic phase, as explained before. Therefore, seasonality should be taken into consideration while sampling and evaluating the results in LWR studies of cephalopods to gain more accurate calculations.

Timing of stop feeding at maturity of many coleoid cephalopods depends on species specific traits^6^. After stop feeding, energy needs of animals are compensated by consuming the energy reservoirs i.e. fat, muscles, digestive gland etc. Therefore, it obviously causes dramatical loss of weight and thus affects the animal’s body condition. Further studies are required to find out possible causes of why mature *S. elegans* males’ coefficient *b* value is slightly higher than that of maturing ones while other investigated species’ vice versa.

Some of the LWR values of Cephalopoda reported had great variances within the same species from different locations and/or authors as discussed above. There could be many factors that might affect LWR calculations that we mentioned in this work need to be investigated in future studies. Different growth type and body structure of cephalopods than fish, calculated *b* values do not indicate the growth type but just could give an idea about the body shape. Cephalopods have different life history traits than many fish species such as semelparity i.e. death after reproduction at the end of rapid growth in relatively short life time (generally around 1 year), might require a different approach to understand their distributions, migrations, weak and strong points of populations in different ecosystems. Therefore, this study aims contributing to knowledge of their biological traits for better assess fisheries managements and invasion biology. Although there were many specimens subjected to this study representing 28 out of 54 defined species from the eastern Mediterranean, some more samples are necessary because of the rarity of some species (e.g. pelagic octopods, some deep-sea species etc.).

## Supplementary Information

Supplementary Information.

## Data Availability

The datasets used and/or analysed during the current study available from the corresponding author on reasonable request.
