# Supplementary material for: Length weight relationships of coleoid cephalopods from the eastern Mediterranean
Source: Sci Rep. 2022 Jul 18;12:12256. doi: 10.1038/s41598-022-16611-7 (PMC9293961; doi:10.1038/s41598-022-16611-7)
Supplement: Supplementary file 1 — Supplementary Information. [file 41598_2022_16611_MOESM1_ESM.docx]

**Length weight relationships of coleoid cephalopods from the eastern Mediterranean**

Bahadır Önsoy and Alp Salman

**Table 2.** LWR data and relevant calculations of 28 eastern Mediterranean cephalopod species (F: Female, M: Male, B: Both sexes, N: Numbers, SE*b*: Standard error of the coefficient *b*, R^2^: Correlation between length and weight, K: Fulton’s condition factor, DML: Dorsal mantle length in centimetres, TW: Total weight in grams, G: Growth pattern, A-: Negative allometry, *A+*: Positive allometry, Iso: Isometry. Mean DML and TW, and their standard deviations are given in parenthesis).

* There were sexually unidentified specimens added to the total number and taken into consideration.

| **Species** | **Sex** | **N** | ***a*** | ***b*** | **SE*b*** | **R^2^** | **K** | **DML (cm)** | **TW (g)** | **G** |
| --- | --- | --- | --- | --- | --- | --- | --- | --- | --- | --- |
|  | F | 398 | 0.185 | 2.875 | 0.059 | 0.96 | 13.84 | 4.4 – 24.1 (11.38±2.74) | 10.60 – 1908.00 (236.87±198.70) | A- |
| *Sepia officinalis* | M | 357 | 0.266 | 2.707 | 0.065 | 0.95 | 13.51 | 6.3 – 21.4 (10.73±2.31) | 35.90 – 1218.50 (184.39±132.32) | A- |
|  | B | 755 | 0.203 | 2.817 | 0.044 | 0.96 | 13.68 | 4.4 – 24.1 (11.07±2.56) | 10.60 – 1908.00 (212.05±172.46) | A- |
|  | F | 761 | 0.279 | 2.401 | 0.051 | 0.92 | 11.67 | 1.9 – 6.6 (4.49±0.79) | 1.00 – 29.30 (10.85±4.38) | A- |
| *Sepia elegans* | M | 720 | 0.356 | 2.159 | 0.060 | 0.88 | 11.28 | 1.9 – 5.9 (4.07±0.64) | 1.42 – 27.50 (7.69±2.82) | A- |
|  | B | 1481 | 0.281 | 2.364 | 0.040 | 0.90 | 11.48 | 1.9 – 6.6 (4.29±0.75) | 1.00 – 29.30 (9.31±4.02) | A- |
|  | F | 969 | 0.271 | 2.558 | 0.032 | 0.96 | 12.95 | 2.2 – 9.4 (5.72±1.48) | 1.60 – 82.90 (26.75±16.56) | A- |
| *Sepia orbignyana* | M | 725 | 0.367 | 2.340 | 0.037 | 0.96 | 12.84 | 2.1 – 9.3 (5.24±1.26) | 1.76 – 60.80 (19.38±10.56) | A- |
|  | B | 1694 | 0.297 | 2.489 | 0.025 | 0.96 | 12.90 | 2.1 – 9.4 (5.52±1.41) | 1.60 – 82.90 (23.59±14.76) | A- |
|  | F | 83 | 0.634 | 2.719 | 0.132 | 0.95 | 45.70 | 1.2 – 6.2 (3.71±1.17) | 1.48 – 94.30 (27.96±22.34) | A- |
| *Rossia macrosoma** | M | 98 | 0.550 | 2.757 | 0.130 | 0.95 | 42.24 | 1.6 – 4.7 (3.28±0.79) | 2.10 – 50.18 (16.71±9.92) | A- |
|  | B | 189 | 0.570 | 2.762 | 0.082 | 0.96 | 44.23 | 1.1 – 6.2 (3.39±1.07) | 0.70 – 94.30 (21.00±17.76) | A- |
|  | F | 226 | 0.549 | 1.777 | 0.192 | 0.60 | 33.37 | 0.8 – 2.3 (1.60±0.31) | 0.19 – 2.73 (1.39±0.54) | A- |
| *Rondeletiola minor* | M | 620 | 0.764 | 1.278 | 0.110 | 0.46 | 33.54 | 1.0 – 2.4 (1.70±0.26) | 0.37 – 2.76 (1.54±0.40) | A- |
|  | B | 846 | 0.670 | 1.488 | 0.098 | 0.52 | 33.50 | 0.8 – 2.4 (1.70±0.28) | 0.19 – 2.76 (1.50±0.45) | A- |
|  | F | 300 | 0.361 | 2.529 | 0.121 | 0.85 | 23.27 | 1.4 – 3.9 (2.68±0.42) | 0.70 – 8.60 (4.62±1.69) | A- |
| *Sepietta oweniana* | M | 505 | 0.663 | 1.924 | 0.138 | 0.60 | 24.07 | 1.7 – 3.5 (2.65±0.31) | 1.14 – 8.46 (4.44±1.22) | A- |
|  | B | 805 | 0.481 | 2.249 | 0.094 | 0.73 | 23.77 | 1.4 – 3.9 (2.66±0.36) | 0.70 – 8.60 (4.50±1.42) | A- |
|  | F | 53 | 0.677 | 2.123 | 0.382 | 0.71 | 43.81 | 1.2 – 2.2 (1.71±0.24) | 0.90 – 3.87 (2.20±0.74) | A- |
| *Sepiola intermedia* | M | 58 | 0.600 | 2.376 | 0.284 | 0.83 | 43.54 | 1.0 – 2.3 (1.77±0.31) | 0.46 – 3.93 (2.48±0.92) | A- |
|  | B | 111 | 0.624 | 2.292 | 0.223 | 0.79 | 43.67 | 1.0 – 2.3 (1.74±0.28) | 0.46 – 3.93 (2.35±0.85) | A- |
|  | F | 4 | 0.705 | 1.742 | 3.623 | 0.68 | 22.47 | 2.4 – 2.8 (2.50±0.20) | 3.03 – 4.24 (3.50±0.58) | **Iso** |
| *Sepiola robusta* | M | 9 | 1.072 | 1.159 | 0.949 | 0.54 | 24.06 | 1.9 – 2.8 (2.30±0.32) | 1.81 – 3.52 (2.86±0.54) | A- |
|  | B | 13 | 0.933 | 1.362 | 0.734 | 0.60 | 23.58 | 1.9 – 2.8 (2.37±0.29) | 1.81 – 4.24 (3.05±0.61) | A- |
|  | F |  |  |  |  |  |  |  |  |  |
| *Sepiola steenstrupiana* | M | 11 | 0.248 | 3.175 | 0.850 | 0.89 | 27.07 | 0.9 – 1.8 (1.42±0.29) | 0.16 – 1.60 (0.86±0.48) | **Iso** |
|  | B |  |  |  |  |  |  |  |  |  |

**Table 2.** (Continued)

| **Species** | **Sex** | **N** | ***a*** | ***b*** | **SE*b*** | **R^2^** | **K** | **DML (cm)** | **TW (g)** | **G** |
| --- | --- | --- | --- | --- | --- | --- | --- | --- | --- | --- |
|  | F | 1074 | 0.068 | 2.638 | 0.032 | 0.96 | 3.24 | 3.4 – 27.8 (9.32±4.14) | 2.31 – 389.20 (38.21±55.35) | A- |
| *Illex coindetii* | M | 794 | 0.040 | 2.948 | 0.039 | 0.97 | 3.67 | 3.5 – 19.7 (9.64±3.74) | 2.08 – 228.30 (49.08±53.74) | A- |
|  | B | 1868 | 0.054 | 2.777 | 0.027 | 0.96 | 3.42 | 3.4 – 27.8 (9.46±3.97) | 2.08 – 389.20 (42.83±54.92) | A- |
| *Ommastrephes bartramii* | F | 15 | 0.008 | 3.406 | 0.219 | 0.99 | 2.96 | 15.7 – 56.0 (26.23±9.74) | 95.00 – 8500.00 (1003.67±2107.01) | ***A+*** |
|  | M | 1 | N/A | N/A | N/A | N/A | 3.20 | 32.0 | 1050.00 | N/A |
|  | B | 16 | 0.008 | 3.405 | 0.205 | 0.99 | 2.98 | 15.7 – 56.0 (26.59±9.52) | 95.00 – 8500.00 (1006.56±2035.60) | ***A+*** |
|  | F | 216 | 0.236 | 2.451 | 0.129 | 0.87 | 8.02 | 4.1 – 14.3 (7.79±1.94) | 7.07 – 137.01 (41.03±27.15) | A- |
| *Todaropsis eblanae* | M | 127 | 0.141 | 2.710 | 0.189 | 0.87 | 7.91 | 5.3 – 14.1 (8.06±1.67) | 8.54 – 131.00 (44.85±26.06) | A- |
|  | B | 342 | 0.203 | 2.523 | 0.106 | 0.87 | 7.99 | 4.1 – 14.3 (7.89±1.85) | 7.07 – 137.01 (42.44±26.72) | A- |
|  | F | 1270 | 0.182 | 2.022 | 0.029 | 0.94 | 4.59 | 1.8 – 9.1 (4.63±1.57) | 0.36 – 18.30 (4.55±3.18) | A- |
| *Alloteuthis media* | M | 1270 | 0.223 | 1.855 | 0.034 | 0.90 | 4.78 | 1.6 – 7.6 (4.18±1.07) | 0.42 – 11.80 (3.58±1.71) | A- |
|  | B | 2540 | 0.194 | 1.966 | 0.022 | 0.92 | 4.69 | 1.6 – 9.1 (4.41±1.36) | 0.36 – 18.30 (3.96±2.62) | A- |
|  | F | 191 | 0.016 | 3.106 | 0.148 | 0.90 | 2.25 | 8.6 – 35 (19.02±3.22) | 46.00 – 1617.80 (173.75±144.24) | **Iso** |
| *Todarodes sagittatus* | M | 114 | 0.008 | 3.367 | 0.170 | 0.93 | 2.32 | 10.5 – 27.6 (18.79±3.40) | 48.00 – 670.00 (177.09±129.29) | ***A+*** |
|  | B | 305 | 0.012 | 3.207 | 0.113 | 0.91 | 2.28 | 8.6 – 35.0 (18.94±3.29) | 46.00 – 1617.80 (174.99±138.63) | ***A+*** |
|  | F | 43 | 0.226 | 2.279 | 0.440 | 0.73 | 8.73 | 2.6 – 4.9 (3.91±0.53) | 1.58 – 9.18 (5.26±1.61) | A- |
| *Abralia veranyi* | M | 33 | 0.156 | 2.644 | 0.573 | 0.74 | 10.37 | 2.3 – 4.0 (3.43±0.47) | 1.00 – 6.22 (4.27±1.36) | **Iso** |
|  | B | 76 | 0.221 | 2.319 | 0.320 | 0.74 | 9.44 | 2.3 – 4.9 (3.70±0.55) | 1.00 – 9.18 (4.83±1.57) | A- |
|  | F | 231 | 0.126 | 2.454 | 0.049 | 0.98 | 3.52 | 4.2 – 25.2 (12.55±5.42) | 3.54 – 385.00 (84.69±81.48) | A- |
| *Loligo vulgaris** | M | 236 | 0.168 | 2.332 | 0.046 | 0.98 | 3.46 | 2.3 – 39.1 (12.85±5.44) | 2.30 – 592.00 (82.02±79.31) | A- |
|  | B | 483 | 0.173 | 2.326 | 0.033 | 0.98 | 3.77 | 1.7 – 39.1 (12.39±5.60) | 1.80 – 592.00 (80.70±80.25) | A- |
|  | F | 218 | 0.185 | 2.395 | 0.035 | 0.99 | 5.18 | 4.0 – 26.5 (9.87±4.97) | 5.00 – 481.00 (66.55±85.22) | A- |
| *Loligo forbesi* | M | 138 | 0.179 | 2.395 | 0.041 | 0.99 | 4.43 | 4.1 – 40.3 (12.89±7.18) | 5.00 – 1250.00 (127.44±186.87) | A- |
|  | B | 356 | 0.186 | 2.388 | 0.026 | 0.99 | 4.89 | 4.0 – 40.3 (11.04±6.10) | 5.00 – 1250.00 (89.54±137.21) | A- |

**Table 2.** (Continued)

| **Species** | **Sex** | **N** | ***a*** | ***b*** | **SE*b*** | **R^2^** | **K** | **DML (cm)** | **TW (g)** | **G** |
| --- | --- | --- | --- | --- | --- | --- | --- | --- | --- | --- |
|  | F | 86 | 0.482 | 2.939 | 0.138 | 0.96 | 44.34 | 3.0 – 27.0 (10.36±5.19) | 10.80 – 6000.00 (848.77±1286.76) | **Iso** |
| *Octopus vulgaris* | M | 152 | 0.467 | 2.986 | 0.107 | 0.95 | 46.76 | 5.4 – 30.0 (13.02±5.09) | 58.70 – 8300.00 (1473.62±1685.59) | **Iso** |
|  | B | 238 | 0.452 | 2.989 | 0.081 | 0.96 | 45.88 | 3.0 – 30.0 (12.05±5.27) | 10.80 – 8300.00 (1247.83±1579.41) | **Iso** |
|  | F | 45 | 0.610 | 2.752 | 0.238 | 0.93 | 38.61 | 2.9 – 11.6 (7.69±2.34) | 13.10 – 516.00 (206.02±144.55) | A- |
| *Octopus salutii* | M | 21 | 2.844 | 2.019 | 0.675 | 0.67 | 39.29 | 5.7 – 9.8 (7.80±1.09) | 88.00 – 375.00 (186.85±67.11) | A- |
|  | B | 66 | 0.678 | 2.705 | 0.214 | 0.91 | 38.83 | 2.9 – 11.6 (7.73±2.02) | 13.10 – 516.00 (199.92±124.94) | A- |
|  | F |  |  |  |  |  |  |  |  |  |
| *Octopus aegina* | M | 15 | 1.069 | 2.269 | 0.949 | 0.67 | 41.49 | 3.2 – 5.2 (3.92±0.71) | 15.00 – 41.00 (25.52±12.27) | **Iso** |
|  | B |  |  |  |  |  |  |  |  |  |
|  | F | 290 | 0.515 | 2.633 | 0.097 | 0.91 | 25.58 | 2.8 – 11.4 (7.51±1.81) | 9.00 – 362.00 (117.94±66.23) | A- |
| *Eledone cirrhosa** | M | 206 | 0.611 | 2.554 | 0.120 | 0.90 | 26.89 | 3.0 – 9.8 (6.85±1.43) | 10.00 – 258.00 (91.56±45.16) | A- |
|  | B | 516 | 0.534 | 2.618 | 0.060 | 0.93 | 26.70 | 1.9 – 11.4 (7.04±1.91) | 1.80 – 362.00 (103.03±61.84) | A- |
|  | F | 366 | 0.892 | 2.397 | 0.101 | 0.86 | 27.18 | 2.9 – 14.3 (8.08±1.93) | 11.50 – 584.00 (150.16±86.26) | A- |
| *Eledone moschata* | M | 278 | 1.570 | 2.142 | 0.140 | 0.77 | 29.96 | 3.4 – 14.8 (7.91±1.98) | 13.50 – 520.00 (147.85±86.83) | A- |
|  | B | 644 | 1.154 | 2.281 | 0.084 | 0.82 | 28.38 | 2.9 – 14.8 (8.01±1.95) | 11.50 – 584.00 (149.16±86.44) | A- |
|  | F | 21 | 3.280 | 2.493 | 0.411 | 0.89 | 148.88 | 2.4 – 8.6 (5.54±1.64) | 28.77 – 854.00 (283.00±207.59) | A- |
| *Pteroctopus tetracirrhus* | M | 23 | 3.966 | 2.258 | 0.269 | 0.94 | 133.63 | 2.5 – 8.5 (4.89±1.61) | 28.00 – 468.75 (164.93±118.14) | A- |
|  | B | 44 | 3.350 | 2.424 | 0.242 | 0.91 | 140.91 | 2.4 – 8.6 (5.20±1.64) | 28.00 – 854.00 (221.29±175.34) | A- |
|  | F | 15 | 1.979 | 2.229 | 0.539 | 0.86 | 79.57 | 1.7 – 5.2 (3.63±0.95) | 5.85 – 61.00 (38.49±19.05) | A- |
| *Scaeurgus unicirrhus* | M | 23 | 1.817 | 2.298 | 0.351 | 0.90 | 84.89 | 2.0 – 5.2 (3.29±0.97) | 6.98 – 91.53 (32.26±22.36) | A- |
|  | B | 38 | 1.872 | 2.272 | 0.274 | 0.89 | 82.79 | 1.7 – 5.2 (3.42±0.96) | 5.85 – 91.53 (34.72±21.08) | A- |
|  | F | 5 | 2.671 | 1.744 | 0.369 | 0.99 | 43.73 | 2.7 – 6.3 (4.72±1.47) | 16.00 – 67.10 (42.30±22.42) | A- |
| *Bathypolypus sponsalis* | M | 1 | N/A | N/A | N/A | N/A | 23.88 | 4.9 | 28.10 | N/A |
|  | B | 6 | 2.727 | 1.684 | 0.811 | 0.89 | 40.42 | 2.7 – 6.3 (4.75±1.32) | 16.00 – 67.10 (39.93±20.88) | A- |
|  | F | 9 | 0.799 | 2.484 | 1.271 | 0.75 | 16.20 | 17 – 33.5 (25.43±5.62) | 765.00 – 5060.00 (2778.67±1522.82) | **Iso** |
| *Ocythoe tuberculata* | M |  |  |  |  |  |  |  |  |  |
|  | B |  |  |  |  |  |  |  |  |  |
|  | F | 5 | 2.999 | 1.940 | 1.785 | 0.80 | 31.52 | 7.5 – 10.3 (8.58±1.25) | 148.90 – 320.00 (199.18±70.20) | **Iso** |
| *Tremoctopus violaceus* | M |  |  |  |  |  |  |  |  |  |
|  | B |  |  |  |  |  |  |  |  |  |
|  | F | 2 | N/A | N/A | N/A | N/A | 47.87 | 3.2 – 3.5 (3.35±0.21) | 16.70 – 19.20 (17.95±1.77) | N/A |
| *Argonauta argo* | M |  |  |  |  |  |  |  |  |  |
|  | B |  |  |  |  |  |  |  |  |  |

**Table 3.** Length – weight relationships of cephalopods from different locations of Mediterranean Sea reported by previous studies and the results of present study (F: females, M: males, B: both sexes).

| *Sepia officinalis* | | | |
| --- | --- | --- | --- |
| **Sex** | ***a*** | ***b*** | **Location and Reference** |
| B | 0.220 | 2.77 | Central Mediterranean; Manfrin Piccinetti and Giovanardi^23^ |
| F | 0.006 | 2.18 | Eastern Mediterranean; Lefkaditou et al.^27^ |
| M | 0.003 | 2.37 |  |
| B | 0.087 | 3.16 | Eastern Mediterranean; Akyol and Metin^26^ |
| F | 0.108 | 2.92 | Eastern Mediterranean; Duysak et al.^28^ |
| M | 0.142 | 2.78 |  |
| B | 0.116 | 2.88 |  |
| F | 0.185 | 2.88 | Present study |
| M | 0.266 | 2.71 |  |
| B | 0.203 | 2.82 |  |
| *Sepia elegans* | | | |
| **Sex** | ***a*** | ***b*** | **Location and Reference** |
| F | 0.257 | 2.51 | Central Mediterranean; Ragonese and Jereb^25^ |
| M | 0.286 | 2.34 |  |
| F | 0.196 | 2.61 | Central Mediterranean; Bello^24^ |
| M | 0.208 | 2.50 |  |
| F | 0.229 | 2.52 | Eastern Mediterranean; Lefkaditou et al.^27^ |
| M | 0.248 | 2.44 |  |
| F | 0.009 | 2.28 | Eastern Mediterranean; Salman^13^ |
| M | 0.003 | 2.15 |  |
| F | 0.279 | 2.40 | Present study |
| M | 0.356 | 2.16 |  |
| B | 0.281 | 2.36 |  |
| *Sepia orbignyana* | | | |
| **Sex** | ***a*** | ***b*** | **Location and Reference** |
| F | 0.657 | 2.15 | Western Mediterranean; Sanchez^31^ |
| M | 0.405 | 2.35 |  |
| F | 0.266 | 2.58 | Central Mediterranean; Ragonese and Jereb^25^ |
| M | 0.272 | 2.48 |  |
| F | 0.224 | 2.56 | Central Mediterranean; Bello^24^ |
| M | 0.208 | 2.56 |  |
| F | 0.343 | 2.31 | Eastern Mediterranean: Lefkaditou et al.^27^ |
| M | 0.525 | 2.44 |  |
| F | 0.271 | 2.55 | Present study |
| M | 0.392 | 2.29 |  |
| B | 0.305 | 2.47 |  |
| *Sepietta oweniana* | | | |
| **Sex** | ***a*** | ***b*** | **Location and Reference** |
| F | 0.225 | 1.61 | Western Mediterranean; Giordano et al.^26^ |
| M | 0.344 | 1.29 |  |
| F | 1.002 | 1.97 | Eastern Mediterranean; Lefkaditou et al.^27^ |
| F | 0.361 | 2.53 | Present study |
| M | 0.663 | 1.92 |  |
| B | 0.481 | 2.25 |  |
| *Todaropsis eblanae* | | | |
| **Sex** | ***a*** | ***b*** | **Location and Reference** |
| F | 0.039 – 0.246 | 2.43 – 3.16 | Western Mediterranean; Belcari et al.^32^ |
| M | 0.163 – 0.680 | 2.11 – 2.64 |  |
| B | 0.141 | 2.70 | Eastern Mediterranean; E. Lefkaditou (in Jereb et al..^9^) |
| F | 0.236 | 2.45 | Present study |
| M | 0.141 | 2.71 |  |
| B | 0.203 | 2.52 |  |

**Table 3.** (continued)

| *Illex coindetii* | | | |
| --- | --- | --- | --- |
| **Sex** | ***a*** | ***b*** | **Location and Reference** |
| F | 0.022 | 3.04 | Western Mediterranean; Belcari^33^ |
| M | 0.011 | 3.39 |  |
| F | 0.002 | 3.02 | Central Mediterranean; Petric et al.^34^ |
| M | 0.016 | 3.45 |  |
| F | 0.030 | 3.00 | Central Mediterranean; Ceriola et al.^35^ |
| M | 0.011 | 3.58 |  |
| F | 0.047 | 2.83 | Eastern Mediterranean; Arvanitidis et al.^36^ |
| M | 0.018 | 3.25 |  |
| F | 0.019 | 3.16 | Eastern Mediterranean; Duysak et al.^28^ |
| M | 0.018 | 3.29 |  |
| F | 0.065 | 2.66 | Present study |
| M | 0.041 | 2.95 |  |
| B | 0.053 | 2.79 |  |
| *Alloteuthis media* | | | |
| **Sex** | ***a*** | ***b*** | **Location and Reference** |
| F | 0.106 | 2.07 | Eastern Mediterranean; E. Lefkaditou (in Jereb et al.^9^) |
| M | 0.143 | 2.01 |  |
| B | 0.244 | 1.76 | Eastern Mediterranean; Akyol and Metin^26^ |
| F | 0.182 | 2.02 | Present study |
| M | 0.223 | 1.86 |  |
| B | 0.194 | 1.97 |  |
| *Todarodes sagittatus* | | | |
| **Sex** | ***a*** | ***b*** | **Location and Reference** |
| F | 0.009 | 3.33 | Western Mediterranean; Quetglas et al.^37^ |
| M | 0.011 | 3.28 |  |
| F | 0.019 | 3.11 | Eastern Mediterranean; E. Lefkaditou (in Jereb et al.^9^) |
| M | 0.008 | 3.39 |  |
| F | 0.016 | 3.11 | Present Study |
| M | 0.008 | 3.37 |  |
| B | 0.012 | 3.21 |  |
| *Loligo vulgaris* | | | |
| **Sex** | ***a*** | ***b*** | **Location and Reference** |
| F | 0.187 | 2.30 | Western Mediterranean; Sanchez^31^ |
| M | 0.121 | 2.50 |  |
| F | 0.197 | 2.45 | Central Mediterranean; Krstulovic Sifner and Vrgoc^38^ |
| M | 0.138 | 2.44 |  |
| F | 0.065 | 2.81 | Eastern Mediterranean; Moreno et al.^39^ |
| M | 0.078 | 2.59 |  |
| B | 0.184 | 2.31 | Eastern Mediterranean; Akyol and Metin^26^ |
| F | 0.019 | 3.16 | Eastern Mediterranean; Duysak et al.^28^ |
| M | 0.018 | 3.29 |  |
| B | 0.002 | 4.08 |  |
| F | 0.126 | 2.45 | Present Study |
| M | 0.168 | 2.33 |  |
| B | 0.173 | 2.33 |  |
| *Loligo forbesi* | | | |
| **Sex** | ***a*** | ***b*** | **Location and Reference** |
| B | 0.141 | 2.46 | Eastern Mediterranean; E. Lefkaditou (in Jereb et al.^9^) |
| F | 0.185 | 2.40 | Present Study |
| M | 0.179 | 2.40 |  |
| B | 0.186 | 2.39 |  |

**Table 3.** (continued)

| *Octopus vulgaris* | | | |
| --- | --- | --- | --- |
| **Sex** | ***a*** | ***b*** | **Location and Reference** |
| F | 0.542 | 2.80 | Western Mediterranean; Guerra and Manriquez^40^ |
| M | 0.350 | 2.99 |  |
| B | 0.420 | 2.92 |  |
| F | 0.413 | 2.92 | Western Mediterranean; Quetglas et al.^41^ |
| M | 0.442 | 2.88 |  |
| B | 0.437 | 2.89 |  |
| F | 1.654 | 2.58 | Western Mediterranean; Sanchez and Obarti^42^ |
| M | 3.306 | 2.32 |  |
| B | 0.51 | 2.87 | Western Mediterranean; Gonzalez et al.^43^ |
|  |  |  |  |
| F | 0.371 | 2.83 | Central Mediterranean; Jabeur et al.^44^ |
| M | 0.485 | 2.83 |  |
| B | 0.399 | 2.92 |  |
| B | 0.138 | 2.60 | Eastern Mediterranean; Lefkaditou et al.^27^ |
|  |  |  |  |
| F | 0.031 | 3.84 | Eastern Mediterranean; Duysak et al.^28^ |
| M | 0.169 | 3.12 |  |
| B | 0.140 | 3.20 |  |
| F | 0.482 | 2.94 | Present Study |
| M | 0.467 | 2.99 |  |
| B | 0.452 | 2.99 |  |
| *Pteroctopus tetracirrhus* | | | |
| **Sex** | ***a*** | ***b*** | **Location and Reference** |
| F | 0.918 | 2.51 | Western Mediterranean; Quetglas et al.^45^ |
| M | 3.283 | 1.88 |  |
| B | 0.681 | 2.62 |  |
| F | 3.280 | 2.49 | Present study |
| M | 5.010 | 2.12 |  |
| B | 3.838 | 2.35 |  |
| *Octopus salutii* | | | |
| **Sex** | ***a*** | ***b*** | **Location and Reference** |
| F | 0.822 | 2.48 | Western Mediterranean; Quetglas et al.^46^ |
| M | 0.807 | 2.47 |  |
| B | 0.758 | 2.51 |  |
| F | 0.610 | 2.75 | Present study |
| M | 2.844 | 2.02 |  |
| B | 0.678 | 2.71 |  |
| *Eledone cirrhosa* | | | |
| **Sex** | ***a*** | ***b*** | **Location and Reference** |
| F | 0.86 | 2.61 | Western Mediterranean; Moriyasu^47^ |
| M | 0.91 | 2.57 |  |
| F | 0.679 | 2.47 | Western Mediterranean; Massi^48^ |
| M | 0.768 | 2.38 |  |
| B | 0.726 | 2.43 |  |
| B | 0.394 | 2.71 | Central Mediterranean; G. Marano (in Jereb et al.^9^) |
| B | 0.336 | 2.28 |  |
| F | 0.56 | 2.56 | Central Mediterranean; Agnesi et al.^49^ |
| M | 1.76 | 1.96 |  |
| F | 1.46 | 2.46 | Central Mediterranean; Giordano et al.^50^ |
| M | 0.56 | 2.15 |  |
| F | 0.841 | 2.51 | Eastern Mediterranean; Lefkaditou et al.^27^ |
| M | 0.915 | 2.43 |  |
| F | 0.515 | 2.63 | Present Study |
| M | 0.611 | 2.55 |  |
| B | 0.534 | 2.62 |  |

**Table 3.** (continued)

| *Eledone moschata* | | | |
| --- | --- | --- | --- |
| **Sex** | ***a*** | ***b*** | **Location and Reference** |
| F | 0.600 | 2.66 | Central Mediterranean; Krstulovic Sifner and Vrgoc^51^ |
| M | 0.525 | 2.77 |  |
| B | 0.323 | 2.70 |  |
| F | 1.119 | 2.23 | Central Mediterranean; Ikica et al.^52^ |
| M | 0.771 | 2.46 |  |
| B | 0.941 | 2.33 |  |
| F | 0.308 | 2.80 | Eastern Mediterranean; Akyol et al.^53^ |
| M | 0.255 | 2.89 |  |
| B | 0.284 | 2.84 |  |
| F | 0.091 | 3.32 | Eastern Mediterranean; Duysak et al.^28^ |
| M | 0.270 | 2.79 |  |
| B | 0.565 | 2.43 |  |
| F | 0.892 | 2.40 | Present Study |
| M | 1.570 | 2.14 |  |
| B | 1.154 | 2.28 |  |
| *Bathypolypus sponsalis* | | | |
| **Sex** | ***a*** | ***b*** | **Location and Reference** |
| F | 0.865 | 2.29 | Western Mediterranean; Quetglas et al.^54^ |
| M | 0.479 | 2.67 |  |
| B | 0.560 | 2.56 |  |
| F | 2.671 | 1.74 | Present study |
| M | N/A | N/A |  |
| B | 2.727 | 1.68 |  |
